# Monotherapy with antibody 1C3 partially protects Ebola virus-exposed macaques

**DOI:** 10.1128/jvi.00296-25

**Published:** 2025-06-10

**Authors:** Gabriella Worwa, Carl W. Davis, Sarah E. Klim, Jacquelyn Turcinovic, Krystle N. Agans, Viktoriya Borisevich, Joan B. Geisbert, Robert W. Cross, Anya Crane, Michael R. Holbrook, Mariano Sanchez-Lockhart, Jeffrey R. Kugelman, Juan A. Patino Galindo, Thomas W. Geisbert, Rafi Ahmed, Jens H. Kuhn, Erica Ollmann Saphire, Gustavo Palacios, Ian Crozier

**Affiliations:** 1 Integrated Research Facility at Fort Detrick, Division of Clinical Research, National Institute of Allergy and Infectious Diseases, National Institutes of Health 650630, Fort Detrick, Frederick, Maryland, USA; 2 Emory Vaccine Center and Department of Microbiology and Immunology, Emory University 197280, Atlanta, Georgia, USA; 3 Galveston National Laboratory, Galveston, Texas, USA; 4 United States Army Medical Research Institute of Infectious Diseases, Fort Detrick, Frederick, Maryland, USA; 5 Icahn School of Medicine at Mount Sinai 5925, New York, New York, USA; 6 Center for Infectious Disease and Vaccine Discovery, La Jolla Institute for Immunology, La Jolla, California, USA; 7 Clinical Monitoring Research Program Directorate, Frederick National Laboratory for Cancer Research, Frederick, Maryland, USA; University of Kentucky College of Medicine, Lexington, Kentucky, USA

**Keywords:** 1C3, 1C11, Ebola virus, EBOV, escape mutation, glycoprotein, monoclonal antibody, orthoebolavirus, partial protection, Sudan virus, SUDV

## Abstract

A cocktail of human monoclonal antibodies 1C3 and 1C11 previously protected macaques from a lethal exposure to either Ebola virus (EBOV) or Sudan virus (SUDV). 1C3 is of particular interest because its paratope strongly binds with unique stoichiometry to the glycoprotein head of several orthoebolaviruses, resulting in neutralization of EBOV and SUDV. Therefore, we evaluated the protective activity of 1C3 as a standalone therapeutic in macaques exposed to either EBOV or SUDV. Two doses of 1C3 monotherapy, administered 4 and 7 days post-exposure, did not protect SUDV-exposed macaques and partially protected EBOV-exposed macaques. Notably, in a macaque that succumbed to EBOV infection, we identified two mutually exclusive escape mutations that emerged immediately after the first dose and resulted in two amino acid changes at the 1C3 binding site. We also detected a subconsensus treatment-emergent mutation likely affecting the 1C3 binding site in all three deceased SUDV-exposed macaques. Our findings highlight combination treatment with 1C11 as critical for protection, particularly against SUDV, and *in vivo* activity of unpartnered 1C3 as susceptible to rapid EBOV and SUDV escape under therapeutic pressure.

**IMPORTANCE:**

A cocktail of human monoclonal antibodies 1C3 and 1C11 previously protected macaques exposed to a lethal dose of either Ebola virus (EBOV) or Sudan virus (SUDV). Since the unique binding characteristics of 1C3 are of particular interest, we evaluated its protective activity as monotherapy in macaques exposed to either EBOV or SUDV. Two doses of 1C3 alone did not protect SUDV-exposed macaques and only partially protected EBOV-exposed macaques. Importantly, failure to protect was associated with the rapid emergence of previously *in vitro*-identified escape mutations at the 1C3 binding site, highlighting the importance of its use in combination with 1C11 for protection against fatal disease outcome and avoiding rapid EBOV and SUDV escape. Findings have broader implications for the wise use of combination-based monoclonal antibody therapeutics to improve outcomes and prevent resistance in filovirid diseases.

## INTRODUCTION

Despite recent progress, improving the outcomes of filovirid diseases caused by Ebola virus (EBOV) and Sudan virus (SUDV) remains an urgent need. Based on the results of the Pamoja Tulinde Maisha (PALM) trial (1), mAb114 (ansuvimab; a single human monoclonal antibody [mAb] that binds to the EBOV glycoprotein [GP_1,2_]) and REGN-EB3 (atoltivimab, maftivimab, and odesivimab; mouse-derived then humanized mAbs that target different epitopes of EBOV GP_1,2_) were approved by the U.S. Food and Drug Administration (FDA) for the treatment of Ebola virus disease (EVD) (2, 3). However, in EVD patients with high viral loads and severe disease, case fatality rates remain high, despite the use of these approved therapeutics (3). Recent outbreaks have highlighted the absence of any clinically evaluated (much less approved) therapeutics for Sudan virus disease (SVD) (4, 5). Encouraging preclinical data in nonhuman primate (NHP) models of SVD has included evaluation of individual or combined mAbs (e.g., MBP134, RIID F6-H2, CA45/FVM04, EBOV-442/EBOV-515) and individual or mAb-combined small-molecule antivirals (e.g., obeldesevir, MBP431/remdesivir) (6–11).

Desirable characteristics to advance candidate mAb therapeutics for EBOV or SUDV include potency, breadth, resistance to viral escape, and ease of administration and production. Indeed, “next-generation” mAb-based therapeutics are ideally cross-neutralizing *in vitro* (against both EBOV and SUDV or even all orthoebolaviruses) (12), are cross-protective *in vivo* (at the lowest dose possible), and have a high barrier to treatment-emergent resistance. The combination of two or three mechanistically independent mAbs, including at different binding sites, is an obvious strategy to optimize and target these goals. However, as demonstrated with mAb114 (for EVD), single-mAb strategies may provide production and dosing advantages and require less complex preclinical evaluation to fulfill acceptance criteria set by regulatory bodies.

Recently, we showed that the combination of two human orthoebolavirus-specific mAbs provided potent protection at low doses against lethal EBOV and SUDV exposure in NHP models (13). mAbs 1C3 and 1C11 dosed at 50 mg/kg (25 mg/kg of each antibody) at 4 and 7 days after exposure were sufficient to protect all macaques from fatal outcome. 1C3 and 1C11 asymmetrically recognize the GP_1_–GP_2_ heterodimers comprising the EBOV GP_1,2_ trimer. While 1C3 interacts with the fusion loop and binds to core epitopes with typical (3:3) stoichiometry, a single fragment antigen-binding region (Fab) of 1C3 binds the GP_1,2_ head region of the virus, where it contacts amino acid (a.a.) residues 115, 117–120, 124, and 172 of all three (1:3) protomers. Previously, based on the targeted 1C3 binding footprint, engineered mutations in EBOV (resulting in GP_1_: K114G, D117A, D117R, S119N, G128R+S46N, R172A, or R172G variants) were shown to result in resistance by *in vitro* assays (13). However, it remains unclear whether these mutations could emerge *in vivo* and impact protection.

To begin to address these questions, we evaluated 1C3 activity against EBOV and SUDV in NHPs. 1C3 was not effective against SUDV in crab-eating macaques but partially protected rhesus monkeys against EBOV. Notably, in samples from one EBOV-exposed animal that suddenly succumbed to disease, we detected the rapid emergence of a.a. changes likely impeding mAb binding sites (at EBOV GP_1_ positions 119 and 172) and enabling viral escape. Moreover, in all three 1C3-treated macaques succumbing to SUDV exposure, we detected a treatment-emergent subconsensus (at >10% of sequences) a.a. change in SUDV GP_1_ position 124. The rapid detection of resistance-associated mutations after treatment sounds a note of caution regarding the use of single-mAb therapeutic strategies for filovirid diseases, particularly for mAbs targeting binding sites otherwise tolerant to structural change that do not impact viral fitness.

## RESULTS

On day 0, four rhesus monkeys were exposed to EBOV and five crab-eating macaques were exposed to SUDV via the intramuscular route using lethal target doses of 1,000 PFU. On day 4 and day 7, 25 mg/kg of 1C3 was administered via intravenous injection to three EBOV-exposed rhesus monkeys (RM 1, RM 2, and RM 3) and three SUDV-exposed crab-eating macaques (CEM 1, CEM 2, and CEM 3). One EBOV-exposed RM was sham-treated with phosphate-buffered saline (RM Control), and two SUDV-exposed crab-eating macaques were not treated (CEM Control 1 and CEM Control 2).

### 1C3 partially protected macaques from death due to EBOV, but not SUDV, exposure

**Fig 1:**
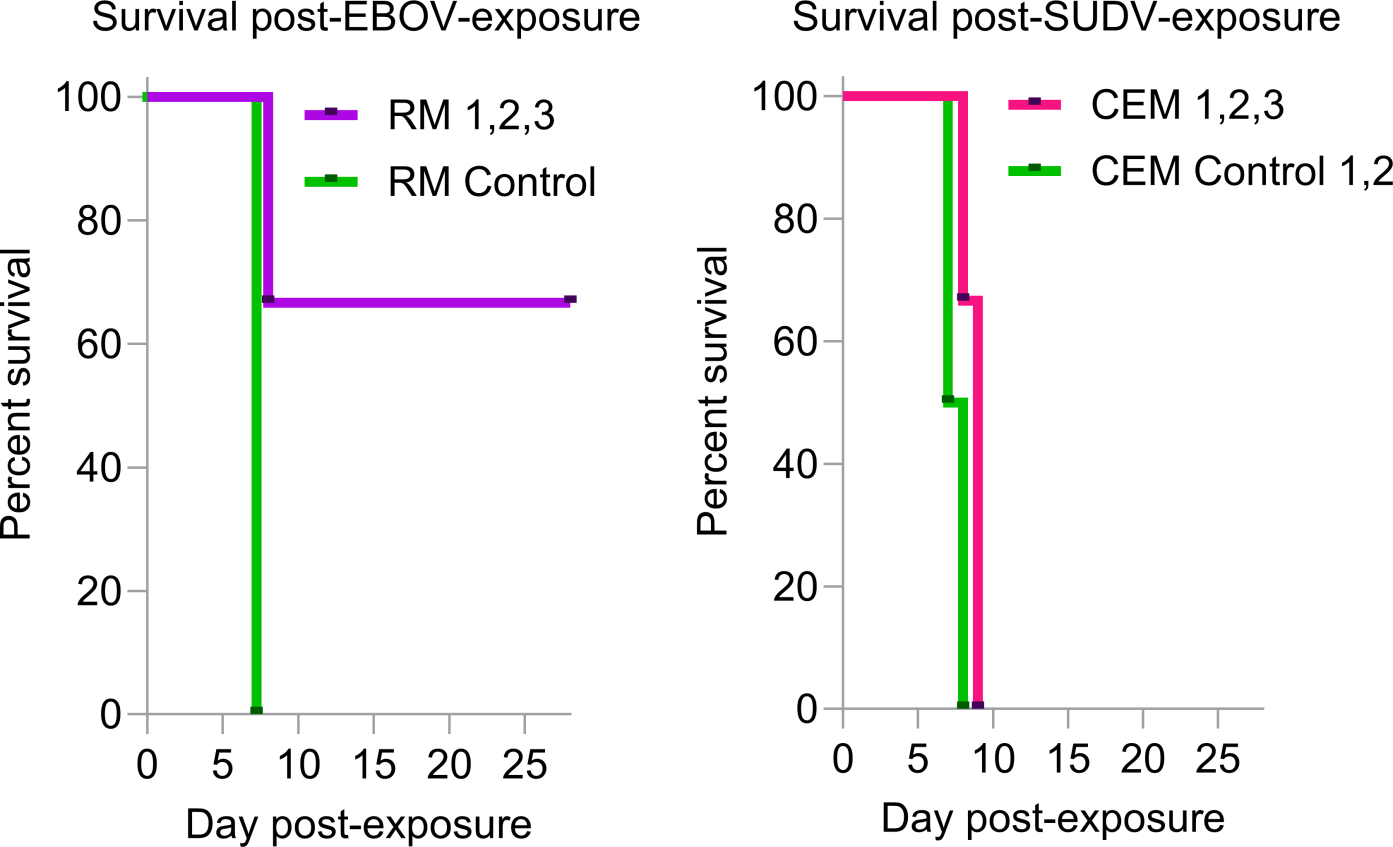
Percent survival of rhesus monkeys and crab-eating macaques after intramuscular exposure on day 0 to EBOV or SUDV, respectively, followed by intravenous injection of 25 mg/kg of 1C3 on day 4 and day 7.

Two of the three EBOV-exposed 1C3-treated rhesus monkeys (RM 1 and RM 2) survived exposure, whereas one 1C3-treated rhesus monkey (RM 3) was found dead on the morning of day 8. The EBOV-exposed sham-treated control rhesus monkey was euthanized on day 8 (Fig. 1). In contrast, none of the crab-eating macaques survived SUDV exposure. The untreated control macaques met euthanasia criteria on day 7 and day 8, whereas the three SUDV-exposed 1C3-treated macaques required euthanasia on day 8 (CEM 2) and day 9 (CEM 1 and CEM 3) (Fig. 1).

### Variable clinical disease severity in 1C3-treated macaques

**Fig 2:**
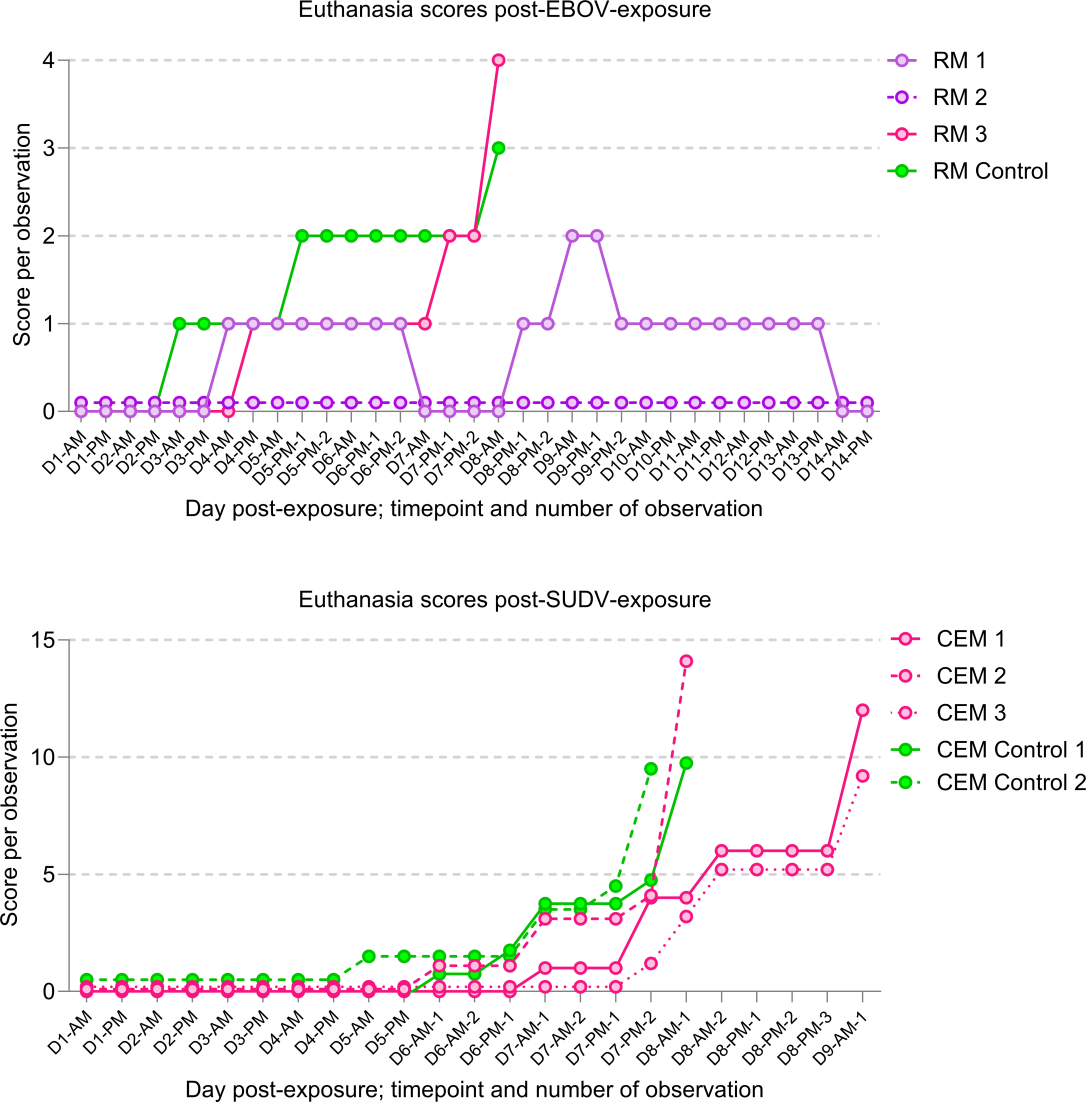
Clinical euthanasia scores recorded for macaques exposed to EBOV or SUDV. Scores are by day (D) and per observation recorded in the morning (AM) or afternoon (PM) and are additionally numbered when more than one observation occurred in the morning or afternoon during the peak disease period. The EBOV scoring scale was 0–4. Scores of 4 or 3, in combination with a rectal body temperature of equal to or less than 34°C, necessitated euthanasia. The SUDV scoring scale was 0–29. Any cumulative scores equal to or above 9 triggered euthanasia. All macaques that met euthanasia criteria or were found dead are shown in magenta; survivors are indicated in purple and controls in green.

Despite small group sizes that limited meaningful group-based statistical comparisons, it is of note that the three EBOV-exposed 1C3-treated rhesus monkeys presented with highly variable clinical signs of disease. Notably, RM 3 began clinically scoring on day 4 (score = 1 for being slightly subdued), progressed on day 7 (score = 2 for withdrawal), then was found dead in cage on day 8 (Fig. 2). In contrast, RM 2 had no clinical signs (score = 0) throughout the experiment. The third macaque in this cohort, RM 1, presented with a prolonged clinical disease course that started on day 4 and lasted until 1 week after administration of the second dose of 1C3 (Fig. 2). This macaque clinically recovered by day 14, but in subsequent weeks developed severe unilateral uveitis that was ultimately associated with persistent vitreal EBOV RNA and severe ocular immunopathology at planned necropsy (day 99) (published previously [14]).

In SUDV-exposed crab-eating macaques, uniform clinical signs and severity were observed in 1C3-treated and control macaques (Fig. 2), although two 1C3-treated macaques met euthanasia criteria 1–2 days later than control animals (Fig. 1 and 2). Hematologic and serum biochemical abnormalities in both experiments were characteristic and well-described previously in these models (Fig. S1 to S4).

### Uncontrolled viremia in 1C3-treated non-survivors

**Fig 3:**
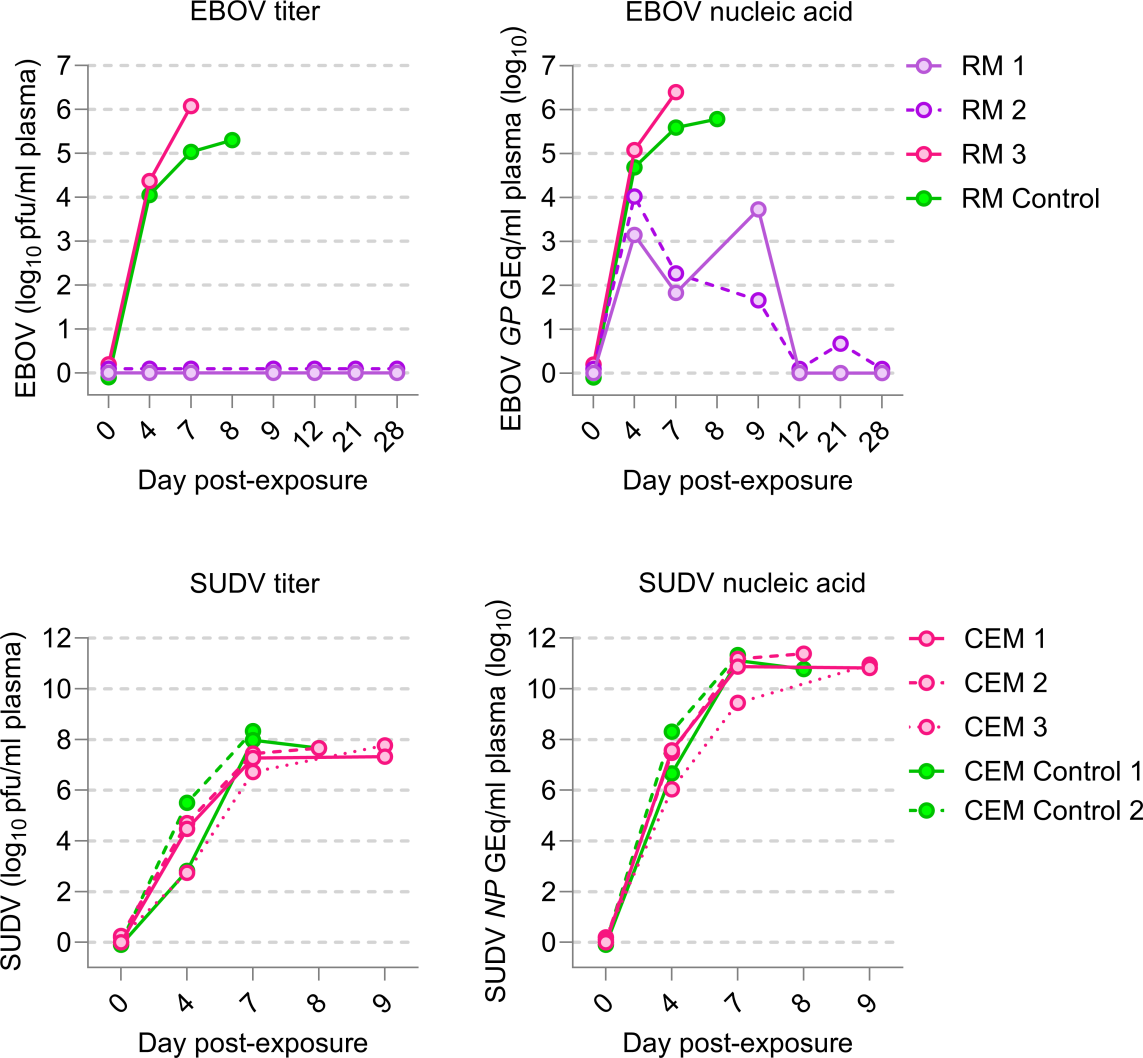
Detectable plasma viremia in macaques over the course of EBOV or SUDV infection. The infectious titers of EBOV and SUDV were determined by plaque assay titration and are shown as PFU per milliliter of plasma. Real-time reverse transcription PCR (RT-qPCR) targeting the glycoprotein gene (*GP*) of EBOV and the nucleoprotein gene (*NP*) of SUDV was used to determine the gene equivalents (GEq) per milliliter of plasma. All macaques that met euthanasia criteria or were found dead are indicated in magenta, survivors are indicated in purple, and controls are indicated in green.

All macaques that succumbed to exposure from day 7 to day 9 had detectable infectious virus in plasma. 1C3 did not limit viral replication in these macaques, as evidenced by control and 1C3-treated non-survivors alike developing EBOV and SUDV viremia with similar kinetics and peak viral RNA loads (Fig. 3). Interestingly, the two EBOV-exposed survivors (RM 1 and RM 2) never had detectable infectious EBOV in plasma, and plasma viral RNA concentrations on day 4 were lower compared to the two EBOV-exposed non-survivors. Although all rhesus monkeys were exposed to the same amount of virus, differences in early EBOV replication (reflected by day 4 EBOV RNA in plasma) likely associate with 1C3’s effectiveness (i.e., lower amounts of virus needed to be neutralized, especially at the lower 25 mg/kg treatment dose). Differences in the day 4 SUDV RNA load were also present in SUDV-exposed crab-eating macaques but did not appear to influence the effectiveness of 1C3, as all 1C3-treated crab-eating macaques developed viremia similar to untreated control crab-eating macaques and eventually succumbed to disease. SUDV viremia peaked on day 7, followed by a plateau until death.

The two EBOV-exposed 1C3-treated survivors (RM 1 and RM 2) cleared viral RNA by day 12 and day 28, respectively (Fig. 3). Interestingly, RM 1, which developed clinical uveitis starting on day 21, presented a biphasic EBOV RNA load pattern, with the first peak detected pre-treatment on day 4 and a second peak on day 9 (Fig. 3).

### 1C3 did not neutralize EBOV from serum collected from RM 3 on and after day 7

To better understand 1C3 treatment failure in one of the three treated EBOV-exposed rhesus monkeys, a virus neutralization assay was performed using sera from RM 3 collected on day 4 and day 7 and tissue homogenates harvested on day 8. A plaque reduction neutralization assay using a 90% neutralization endpoint demonstrated complete neutralization in day 4 serum, no neutralization in day 7 serum, and only partial neutralization in day 8 tissue homogenates.

### Escape variants identified within the 1C3 binding site of the EBOV and SUDV glycoproteins

**Fig 4:**
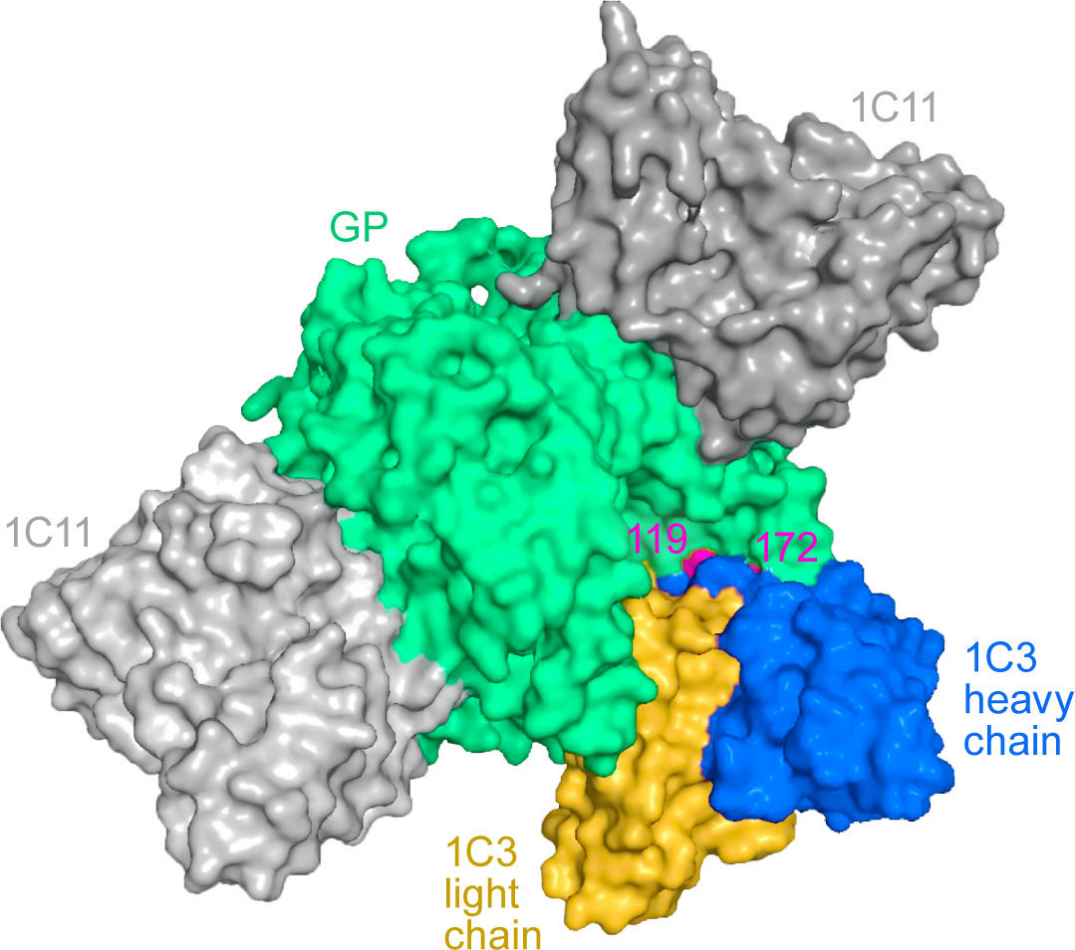
Structure of the EBOV glycoprotein (GP_1,2_; green) with 1C3 light chain (yellow) and heavy chain (blue). Residues 119 and 172 (red) undergo substitutions S119N and R172Q. Binding of antibody 1C11 (gray) is shown for reference.

**Fig 5:**
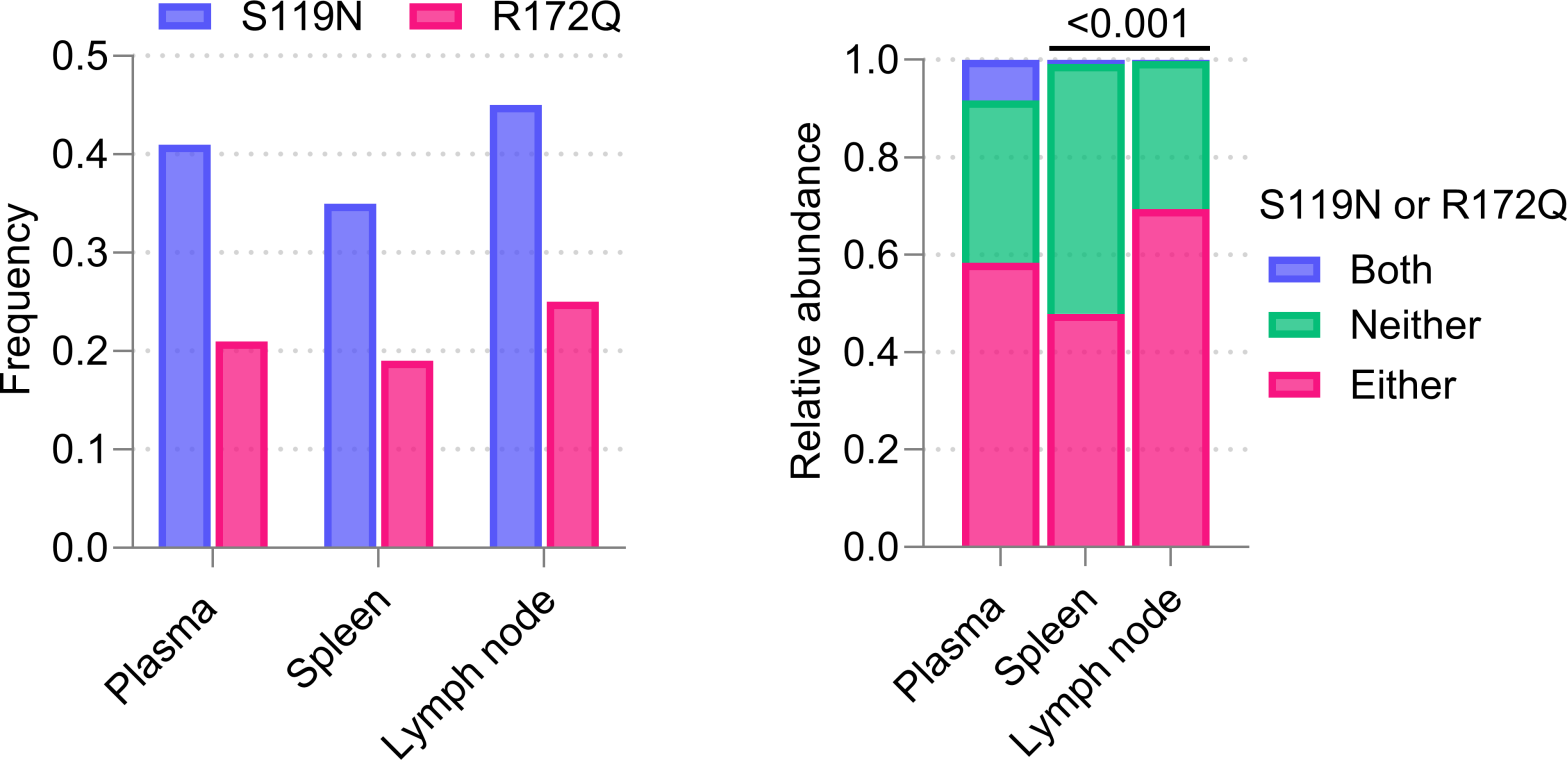
Left: frequency of G-to-A mutations at nucleotide positions 6,393 and 6,552 leading to amino acid substitutions S119N and R172Q in sequences of EBOV from plasma collected on day 7 and spleen and tracheobronchial lymph node of RM 3 harvested on day 8. Right: relative abundance (as a ratio of 1.0) suggesting exclusivity or coexistence of S119N and R172Q in the same sample type. Based on Fisher’s exact test, a significant difference (*P* < 0.001) was found in the abundance of either S119N or R172Q in spleen and lymph node.

Viral genome sequencing was conducted using nucleic acid from plasma and tissues collected from 1C3-treated EBOV-exposed RM 3. As compared to the inoculum (which served as the reference for variant calling), several nucleic acid mutations were identified (Table S1). Notably, there were two G-to-A mutations in the *GP* gene region at nucleotide positions 6,393 and 6,552, corresponding to GP_1_ a.a. positions 119 and 172, respectively, and leading to S119N and R172Q a.a. substitutions. Among samples, the detection frequency of S119N and R172Q varied, with an abundance of up to 45% in day 7 plasma and spleen and lymph node tissues but a complete absence in liver tissues (Table S1). Both EBOV GP_1_ a.a. changes are located within and interact with the binding site of 1C3 (Fig. 4) and had been previously identified in *in vitro* resistance assays (13). Across samples, S119N and R172Q detection frequency (Fig. 5) was nearly mutually exclusive, with detection of either substitution only in independent sequences.

**Fig 6:**
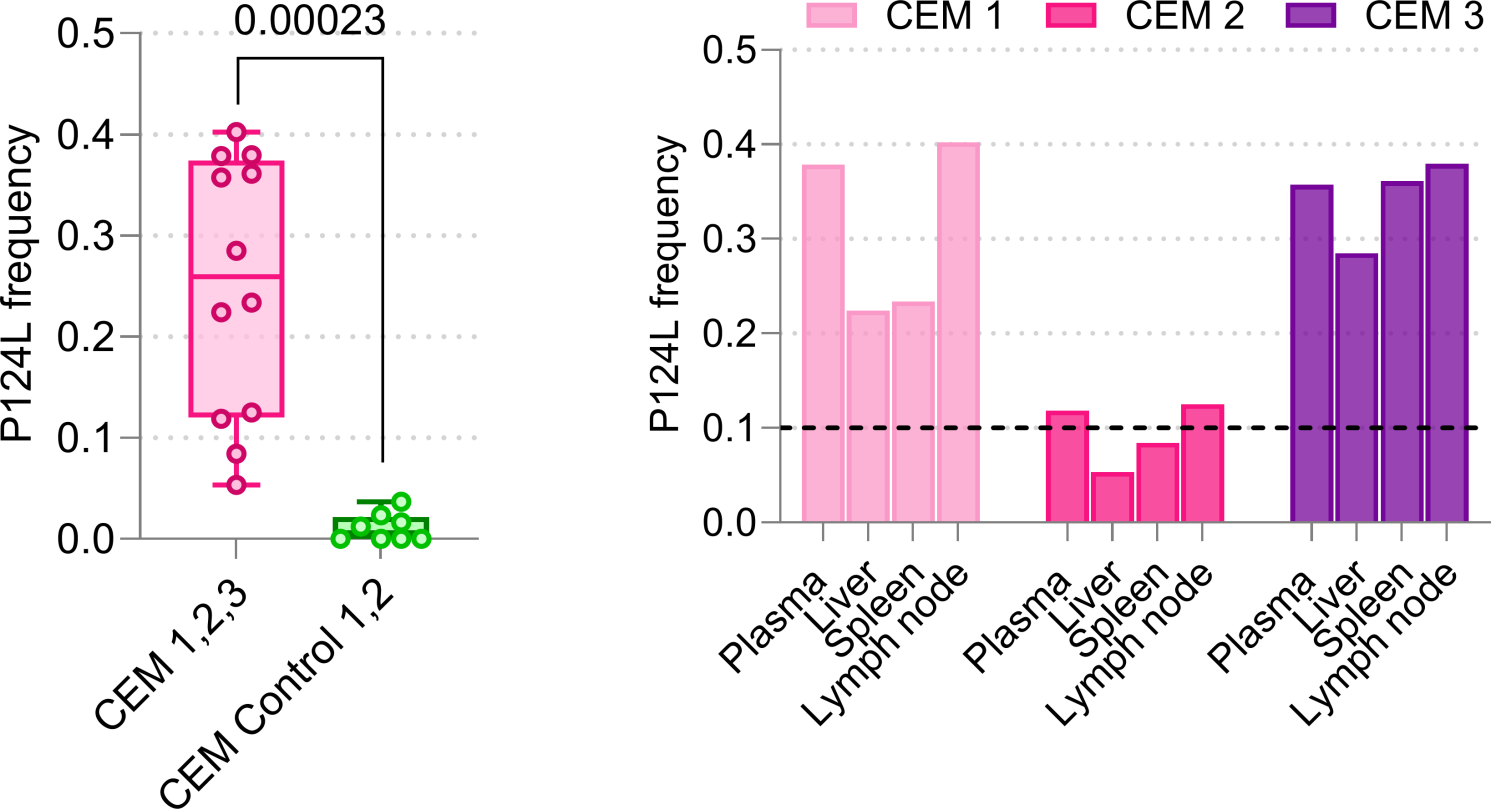
Left: frequency of amino acid change for P124L in SUDV GP_1,2_ was significantly (*P* < 0.001) higher in plasma and tissue homogenates from 1C3-treated macaques (magenta) than from untreated controls (green). Right: change in P124L was detectable at a frequency of 0.1–0.4 in plasma, spleen, liver, and axillary lymph node from CEM 1 and CEM 3. In CEM 2, GP_1_ P124L was also detectable in all tissues but at >0.1 frequency (black dashed line) in plasma and lymph node only.

To test whether treatment with 1C3 generated resistance mutations in the SUDV exposure study, we analyzed viral genomes from three crab-eating macaques (CEM 1, CEM 2, and CEM 3) and two controls. We identified one non-synonymous mutation (leading to a substitution at GP_1_ P124L), present in >10% of viral genomes in at least one tissue in all three SUDV-exposed macaques (Fig. 6). In contrast, this mutation was either undetectable or present in <4% of viral genomes in control macaques (*P* < 0.001).

## DISCUSSION

Our findings suggest that unpartnered 25 mg/kg doses of 1C3 administered on day 4 and day 7 are not fully effective post-exposure therapy in lethal models of EVD and SVD. Partial protection of EBOV-exposed rhesus monkeys (versus no protection of SUDV-exposed crab-eating macaques) suggests differential activity of 1C3 in these models that could be influenced either by the specific 1C3–orthoebolavirus interaction (more likely) at this dose or alternatively (less likely) may be related to different NHP host species, or both.

After two 25 mg/kg doses of 1C3 on day 4 and day 7, an EBOV-exposed rhesus monkey was found dead on day 8; we identified GP_1_ amino acid (a.a.) substitutions S119N and R172Q in day 7 plasma and in day 8 spleen and tracheobronchial lymph nodes. Because 1C3 binds to these a.a. positions on all three GP_1,2_ monomers (13), positions 119 and 172 are key determinants of mAb binding. Previously, we showed that sub-neutralizing concentrations of 1C3 led to mutations resulting in changes in EBOV GP_1_ at a.a. positions 117, 118, 119, and 128 (13), and S119N, R172A, and R172G were associated with pseudoviral escape *in vitro* (13). In that study, a.a. changes at position 172, specifically R172A and R172G, were not neutralized by 1C3 *in vitro*, suggesting that the R172Q a.a. change detected newly here might also have impacted neutralization by 1C3 and potentially contributed to the fatal outcome of this macaque.

We identified SUDV GP_1_ a.a. change P124L with a frequency of >10% in at least two specimens from all three 1C3-treated SUDV-exposed macaques, which succumbed on day 8 and day 9 after two treatment doses. SUDV GP_1_ P124 is one of five non-conserved residues within the 1C3 binding footprint (13). In EBOV GP_1_, residue A124 on all three protomers physically contacts 1C3. Crucially, GP_1_ 124 occupies a geometrically lower position in the glycoprotein chalice than the three residues critical for Niemann-Pick C1 (NPC1) binding to EBOV GP_1_ (15), two of which (K114 and K115) are also part of the 1C3 footprint. Plausibly, GP_1_ P124L could contribute to viral escape from neutralization at a minimal viral fitness cost to receptor binding.

The rapid emergence of EBOV GP_1_ resistance within just 3 days of the administration of unpartnered 1C3 is concerning. First, it indicates that EBOV and SUDV, under selective pressure, can adapt rapidly at this binding site, potentially compromising neutralization (certainly the case for RM 3 in this study) and therapeutic effectiveness as well as reducing the effective therapeutic window. Second, it emphasizes the need to carefully evaluate the tolerance to a.a. changes at mAb binding sites; some filovirid GP binding sites are less prone to change due to structural and/or fitness constraints, making them more suitable targets for therapeutic mAbs. For example, mAb114’s mechanism of neutralization and binding target includes the receptor binding domain and an essential receptor binding step (at a location less tolerant to change for viral fitness costs). Indeed, samples sequenced during extensive human-to-human transmission during the Western African EVD outbreak showed near 100% conservation of the mAb114 GP_1_ epitope. The absence of plasticity related to a fitness cost may limit the development of resistance even when used as monotherapy (2, 16, 17). However, more structurally tolerant mAb binding sites, in which a.a. changes do not have significant fitness costs, may be susceptible under selective therapeutic pressure to rapid escape, as seen after 1C3 in the sera of RM 3.

1C3 was administered at a relatively low dose (25 mg/kg) compared to doses typically given with approved mAb therapeutics. We speculate that higher doses of 1C3 may reliably protect macaques and prevent viral escape after lethal EBOV exposure. However, the combination of 1C3 and 1C11 is likely to provide not only superior therapeutic effectiveness but also protection from treatment-emergent resistance; indeed, *in vitro* investigation of this combination demonstrated synergy between 1C3 and 1C11 (combination index of 0.63 against EBOV) and full neutralization of escape mutations to one mAb by the other mAb component (13). Combining 1C3 with 1C11 for treatment of EBOV-exposed guinea pigs previously improved survival compared to 1C3 monotherapy, but the combination was not evaluated in SUDV-exposed guinea pigs. Both S119N and R172Q a.a. changes were found with up to 40% abundance in samples of plasma, spleen, and tracheobronchial lymph nodes (Table S1), notably independently, suggesting mutually exclusive emergence (Table S2). The detection of a varying frequency of mutations among different tissues in this macaque suggests tissue site-specific viral evolution in the presence of 1C3; the complete absence of S119N or R172Q detection in EBOV RNA sequenced from liver tissue of RM 3 implies either varying selection pressure at these sites or an organ-specific distribution of 1C3. In general, the relative tissue penetration of large mAb molecules is plausibly much less than in the systemic circulation (18); however, definitive determination of biodistribution is experimentally very challenging. We speculate that the identified mutations would revert to the wild-type sequence absent selective pressure by 1C3, though these mutations might afford an independent viral fitness benefit. However, without plausible evidence that these specific mutations could increase GP_1_ receptor binding efficiency, attachment, and viral replication or virulence, we find this unlikely.

The relationship between mAb-based therapeutics and the development of post-acute sequelae of EVD, including viral persistence, remains largely unexplored. In the context of late recrudescent or relapsed EVD in a survivor previously treated with mAb114, EBOV GP_1_ mutations were detected but were distant from the mAb114 binding site, and mAb114 retained neutralization activity in pseudovirus assays (19). EVD-associated uveitis is frequently reported in EVD survivors (20, 21) and has been associated with EBOV persistence in intraocular fluid (22). Interestingly, the 1C3-treated EBOV-exposed survivor (RM 1) later developed a destructive unilateral panuveitis associated with EBOV RNA detection in the ocular vitreous sampled 99 days after exposure, as previously described (14). It is possible that prolonged acute clinical disease associated with biphasic viral load peaks and delayed clearance of EBOV (Fig. 2) enabled seeding of this immune-privileged site in this animal. Whether these viral dynamics were associated with viral resistance and/or partial escape cannot be determined; sequencing of later specimens from this rhesus monkey was not possible. Though not after mAb monotherapy, “atypical” late disease in an NHP after treatment with mAbs CA45 and FVM04 has been associated with the emergence of a single EBOV GP fusion loop mutation (E545D) that led to resistance (to the mAb CA45 component), increased viral growth in cell culture, and possibly increased virulence in domestic ferrets (23). In contrast to our study’s 1C3-treated rhesus monkeys with sudden death (RM 3) or biphasic prolonged acute viremia and late uveitis (RM 1), the third macaque (RM 2) never developed clinical signs and recovered unremarkably. Indeed, the acute and post-acute features after EBOV exposure and 1C3 treatment in these three rhesus monkeys varied widely.

Viral resistance to immunotherapies has been described, though typically over an extended period. For example, human immunodeficiency virus 1 resistance to mAb VRC01 monotherapy appears over weeks (in one study, at a median of 39 days) (24, 25). Similarly, influenza A virus can develop resistance to mAbs, but it requires multiple replication cycles and a combination of selective pressures over time (26). More recent experience in the coronavirus disease 2019 pandemic, however, has identified earlier treatment-emergent resistance to mAbs targeting severe acute respiratory syndrome coronavirus 2, including as early as 6 days after treatment (27, 28). Our *in vivo* findings suggest that reliance on single neutralizing mAbs for filorivid disease carries a risk of rapid treatment-emergent-resistant mutations that increase the likelihood of viral escape and treatment failure. In the context of prior demonstration of protection by combined mAbs 1C3 and 1C11 (against both EBOV and SUDV lethal exposures), our results support the use of multiple mechanistically independent orthoebolavirus GP-specific mAbs to mitigate treatment-emergent resistance in addition to improving therapeutic success and patient outcomes. Recent investigation of already-approved therapeutics (REGN-EB3 and mAb114) for EVD has shown that component mAbs, even those directed against conserved viral epitopes, are susceptible to the rapid development of escape mutants *in vitro* when used as monotherapy; escape is robustly prevented by mAb combination (29).

We acknowledge several limitations. First, the small number of macaques (*n* = 3 per group) in this exploratory study limits meaningful group-based statistical comparisons. Second, observations were limited by experimental design and NHP constraints; i.e., the absence of a single-dose (versus two-dose) mAb comparator somewhat limits the interpretation of results. Challenges intrinsic to the biosafety level 4 (BSL-4) setting precluded determination of concentrations of 1C3 in specific tissues. In addition, the comparison of protection and virus evolution between the two orthoebolaviral exposures, even after the same 1C3 dosing regimen, could be impacted by experimental design, including dose and timing of treatment, host features in different macaque species, and facility-specific variables. With regard to the GP_1_ R172Q a.a. substitution, though plausible based on prior *in vitro* identification of resistance at this location (R172A, R172G) (29), we have not specifically interrogated the R-to-Q change in pseudovirus assays as further experiments on the replicative fitness of the identified mutants were beyond the scope of this study. Finally, our deep-sequencing analysis was inclusive of samples from only one EBOV-exposed macaque (RM 3), and it is possible that the same or other escape mutations were present in one or several of the other macaques.

In conclusion, 1C3, dosed at 25 mg/kg, administered 4 and 7 days after exposure, partially protected EBOV-exposed macaques and did not protect SUDV-exposed macaques. Building on previously published *in vitro* data, our findings suggest that unpartnered 1C3 treatment of filovirid-exposed macaques can lead to the rapid emergence of escape mutations *in vivo* that may be associated with treatment failure and have broader implications for the wise use of combination mAb-based strategies for filovirid diseases.

## MATERIALS AND METHODS

### Nonhuman primate studies

NHP exposures to EBOV were performed at the NIAID IRF-Frederick, and exposures to SUDV were performed at UTMB, as previously described (13). Briefly, macaques of Chinese origin of both sexes (WorldWide Primates, Miami, FL, USA) were assigned to the study. On day 0, four rhesus monkeys [*Macaca mulatta* (Zimmermann, 1780)] were exposed intramuscularly to 1,000 PFU of EBOV (Ebola virus/H.sapiens-tc/COD/1995/Kikwit-9510621; NR-50306, Lot 9510621; BEI Resources, Manassas, VA, USA), and five crab-eating macaques (*Macaca fascicularis* Raffles, 1821) were exposed intramuscularly to 1,000 PFU of SUDV (supernatant originated from the second of two passages of Sudan virus/H.sapiens-tc/UGA/2000/Gulu-808892 inoculated onto Vero E6 cells (CRL-1586; American Type Culture Collection [ATCC], Manassas, VA, USA) at a multiplicity of infection of 0.001 and harvested 7 days later). On day 4 and day 7, three virus-exposed rhesus monkeys and three crab-eating macaques were administered 25 mg/kg of human 1C3 intravenously (anti-EBOV GP_1,2_ 1C3; Zalgen Labs, Frederick, MD, USA). One rhesus monkey and two crab-eating macaques served as virus-exposed controls and were either sham-treated with phosphate-buffered saline or not treated after EBOV and SUDV exposures, respectively. All macaques were observed daily at cageside. Sedations were performed at 4, 7, 9, 12, 21, and 28 days for EBOV-exposed macaques (at the IRF-Frederick), and at 4 and 7 days for SUDV-exposed macaques (at UTMB). Physical examinations and collection of venous blood were performed during each sedation and prior to euthanasia. Clinical euthanasia scoring differed by location. EBOV-exposed macaques (at the IRF-Frederick) were assigned daily scores: normal activity and responsiveness (0), slightly subdued (1), withdrawn (2), temporarily recumbent (3), or persistently recumbent (4); scores of 4 or 3, in combination with a rectal body temperature ≤34°C, required euthanasia. SUDV-exposed macaques (at UTMB) were assigned daily scores in the categories of respiration, appetite, activity, appearance, and bleeding. Any scores equal to or above 9 triggered euthanasia.

### Hematology and serum chemistry

Whole blood from EBOV-exposed macaques was analyzed on a XT-2000iV hematology instrument (Sysmex America, New York, NY, USA) to obtain a complete blood count with leukocyte differential. Whole blood from SUDV-exposed macaques was assessed using a laser-based hematologic analyzer (Beckman Coulter, Brea, CA, USA). Plasma and sera from virus-exposed macaques were obtained following centrifugation at 1,800 × *g*. Serum chemistry for EBOV-exposed macaques was assessed using the Piccolo general chemistry 13 panel on a Piccolo Xpress analyzer (Abaxis, Parsippany, NJ, USA) and included ALT, albumin, ALP, amylase, AST, blood urea nitrogen (BUN), calcium, creatinine, gamma-glutamyl transferase (GGT), glucose, total bilirubin, total protein, and uric acid. Serum chemistry for SUDV-exposed macaques was assessed using Biochemistry Panel Plus disks (Abaxis) on a Piccolo point-of-care analyzer and included ALT, albumin, ALP, amylase, AST, BUN, calcium, C-reactive protein, creatinine, GGT, glucose, total protein, and uric acid.

### Nucleic acid isolation and real-time reverse transcription PCR (RT-qPCR)

Viruses in serum samples from EBOV-exposed macaques were inactivated by immersion in TRIzol LS in accordance with the manufacturer’s instructions (Thermo Fisher Scientific, Waltham, MA, USA). Nucleic acid was extracted with the QIAamp Viral RNA Mini Kit (QIAGEN, Hilden, Germany). Downstream EBOV *GP* gene detection was performed by RT-qPCR using the BEI Resources Critical Reagents Program EZ1 RT-PCR kit assay in accordance with the manufacturer’s instructions (30), and analysis was conducted on an Applied Biosystems 7500 FastDx Real-Time PCR instrument (Thermo Fisher Scientific). Blood samples from SUDV-exposed macaques were inactivated in AVL viral lysis buffer prior to removal from the BSL-4 laboratory, and nucleic acids were isolated using the QIAamp Viral RNA Mini Kit (QIAGEN) in accordance with the manufacturer’s recommendations. RT-qPCR assays for the detection of the SUDV *L* gene were performed on a CFX96 detection system (Bio-Rad Laboratories, Hercules, CA, USA), as described previously (13). Results were plotted as genome equivalents per milliliter.

### Plaque assay titration

Titers of infectious EBOV in plasma were determined by plaque assay titration as described previously (31). Briefly, plasma samples were diluted 10-fold in Gibco Dulbecco’s Modified Eagle Medium (DMEM; Thermo Fisher Scientific), adsorbed on 24-hour-old monolayers of grivet (*Chlorocebus aethiops* (Linnaeus, 1758)) Vero E6 cells (CRL-1586; ATCC), seeded in six-well plates, and incubated at 37°C and 5% carbon dioxide (CO_2_) for 1 h. After incubation, 2 mL of a 2.5% Avicel (FMC Biopolymer, Philadelphia, PA, USA) overlay was added to each well, and plates were incubated at 37°C and 5% CO_2_ for 7 days. Avicel was removed, and wells were covered with 2 mL of a 0.2% crystal violet stain and incubated at ambient temperature for 30 min. Then, plates were rinsed with tap water and air-dried. Plaque numbers were counted and calculated per milliliter of sample. Titers of infectious SUDV were determined by a previously described plaque assay titration protocol (1) using confluent monolayers of Vero E6 cells.

### Plaque reduction neutralization assay

An endpoint neutralization assay was run in duplicate on diluted samples from one EBOV-exposed non-surviving macaque (RM 3) using confluent Vero E6 cells. Sera collected on day 4 (prior to first 1C3 administration) and on day 7 (prior to second 1C3 administration), homogenates from livers and pancreases collected on day 8 (after administration of both 1C3 doses), and an EBOV stock (serving as positive control) were diluted to a concentration of 150 PFU in 200 µL of phosphate-buffered saline. Serum and homogenate samples were mixed with 25, 50, 100, or 200 µg/mL of 1C3 using equal volumes, and EBOV Kikwit stock was mixed 1:2 with DMEM. Mixed samples were incubated at 37°C for 1 h and transferred onto Vero E6 monolayers seeded in six-well plates. All subsequent steps followed the plaque assay titration protocol. The number of plaques was counted, and neutralization of samples qualitatively assessed using a 90% neutralization endpoint (PRNT_90_) based on the number of plaques generated by the EBOV positive control.

### Full-genome sequencing and variant analysis

#### Library preparation

Plasma collected on day 7 and tissue homogenates generated from liver, spleen, and tracheobronchial lymph node specimens collected on day 8 from EBOV-exposed RM 3 were used for viral genome sequencing with a targeted enrichment protocol with probes designed against EBOV (GCA_000848505.1). Briefly, RNA was extracted using the PureLink RNA mini kit (Thermo Fisher Scientific). RNA sequencing libraries were prepared using the TruSeq RNA LP for Enrichment kit (Illumina, San Diego, CA, USA) with xGen Dual Index UMI Adaptors (Integrated DNA Technologies, Skokie, IL, USA) in accordance with the manufacturers’ guidelines. A total of 20 ng of exogenous human cervical adenocarcinoma (HeLa) cell Total RNA Control (Thermo Fisher Scientific) was spiked into each RNA sample, and then RNA was fragmented at 94°C for 0.5–2 min, depending on sample RNA quality. After library preparation, TruSeq RNA Exome enrichment reagents (using one-quarter quantities to enable singleplex enrichment) (Illumina) were used with a custom biotinylated probe set. For singleplex enrichment, the quantity of the reagents was reduced to one-quarter of manufacturer’s recommendations.

Viral genome sequencing was performed on terminal plasma samples and homogenates from spleen, liver, and axillary lymph node specimens from SUDV-exposed CEM 1, CEM 2, and CEM 3. Tissue homogenization and isolation of RNA from samples was performed as described previously (13). All RNA samples were cleaned, and DNA was digested with the Zymo Clean and ZR-96 Genomic DNA Clean & Concentrator-5 kit (Zymo Research, Tustin, CA, USA). Sequencing libraries were constructed after rRNA depletion and sequenced on an Element Aviti System Sequencing instrument (Element Biosciences, San Diego, CA, USA) to a target depth of 50 million reads per sample.

#### Sequencing, sequence processing, and reference alignment

All samples from EBOV-exposed macaques were run on a MiSeq instrument (Illumina). Preprocessing of reads was completed using a combination of PRINSEQ-lite (32) and in-house-developed cleaning scripts. For analysis, paired FastQ files were aligned and compared to the reference virus (inoculum). Duplicates were removed using Picard (33), and variants called using iVAR software (34). Illumina sequencing did allow phase analyses once mutations of interest were observed at GP_1_ a.a. 119 and 172 (BEI reference nucleotide positions 6,552 and 6,393, respectively) as close enough to be present in the same sequencing read. Reads mapping to the reference that included both positions were selected, and a Fisher’s exact test was performed to assess the co-occurrence of these mutations in the same read on each sample.

Host reads obtained from SUDV-exposed macaques were removed by aligning to the *M. fascicularis* v.6.0 genome assembly using Bowtie 2 v.2.4.21 with the very-sensitive flag (35). Reads that did not align to the crab-eating macaque genome were then aligned to the SUDV Gulu reference sequence (NCBI accession NC_006432.1) with Bowtie 2. The SAMtools library v.1.182 was used to process alignment files and evaluate coverage (36). Single nucleotide variants (SNVs) were called with LoFreq v.2.1.3.13 (37) and annotated with a custom R script using the Biostrings library (38). The glycoprotein coding sequence had >100 aligned reads per nucleotide in all samples. Potential adaptive SNVs were identified by filtering for non-synonymous *GP* SNVs present at >10% in treated macaques but not controls. Calculation of *P*-values was done using the Wilcoxon nonparametric test.

## Data Availability

All raw data are available upon request. SUDV genomic analysis code is available on GitHub (https://github.com/geisbert-lab/cyno-sudv-prot1902012-1). SUDV sequencing data are available under BioProject accession number PRJNA1135922.

## SUPPLEMENTAL MATERIAL

Supplemental material jvi.00296-25-s0001.pdf Tables S1 and S2; Figures S1 to S4.

ASM does not own the copyrights to Supplemental Material that may be linked to, or accessed through, an article. The authors have granted ASM a non-exclusive, world-wide license to publish the Supplemental Material files. Please contact the corresponding author directly for reuse.
