# Supplementary material for: Monotherapy with antibody 1C3 partially protects Ebola virus-exposed macaques
Source: J Virol. 2025 Jun 10;99(7):e00296-25. doi: 10.1128/jvi.00296-25 (PMC12282081; doi:10.1128/jvi.00296-25)
Supplement: Supplemental material — Tables S1 and S2; Figures S1 to S4. [file jvi.00296-25-s0001.pdf]

SUPPLEMENTARY TABLES

Supplementary Table 1 Ebola virus (EBOV) variant calling in sequences of samples collected from RM 3. Mutations leading to amino acid (a.a.) changes S119N and R172Q and corresponding frequences are written in bold magenta font.

**Mutation (resulting a.a. substitution)**	**Gene**	**Protein Position**	**Inoculum[1] Day 0**	**Plasma Day 7**	**Liver Day 8**	**Spleen Day 8**	**Lymph node Day 8**
2026 (R→G)	*NP*	519	N/A	0.29	0	0	0
5076 (N→S)	*VP40*	200	N/A	0	0	0	0.25
5201 (T→A)	*VP40*	242	N/A	0	0	0	0.1
6266 (T→A)	*GP*	77	N/A	0	0	0	0.03
6386 (D→N)	*GP*	117	N/A	0	0	0.05	0.06
6387 (D→G)	*GP*	117	N/A	0	0	0	0.04
6392 (S→G)	*GP*	119	N/A	0	0	0.05	0
**6393 (S→N)**	***GP***	**119**	N/A	**0.41**	0	**0.35**	**0.45**
6396 (G→R)	*GP*	120	N/A	0	0	0.03	0
6419 (G→R)	*GP*	128	N/A	0	0	0.03	0
**6552 (R→Q)**	***GP***	**172**	N/A	**0.21**	0	**0.19**	**0.25**

6932 (R→K)	*GP*	299	N/A	0	0	0	0.04
7667 (I→T)	*GP*	543	019	0	0	0.12	0.05
11586 (T→A)	*L*	3	N/A	0	0	0.06	0

[1]The inoculum administered to macaques on Day 0 was used as the reference stock for variant calling.

Supplementary Table 2 Number of G-to-A mutations at nucleotide positions 6393 and 6552 (leading to a.a. substitutions S119N and R172Q) in sequences of Ebola virus (EBOV) from plasma collected on Day 7, and spleen and tracheobronchial lymph node of RM 3 harvested on Day 8. Bottom rows show the number of both, either or neither mutation occurring within the same sample type.

**Mutation**	**Plasma**	**Spleen**	**Lymph node**	**Total**
**6393 (S→N)**	12	66	323	401
**6552 (R→Q)**	2	46	184	232
Both	2	2	2	6
Either	14	112	507	633
Neither	8	120	222	350

SUPPLEMENTARY FIGURES

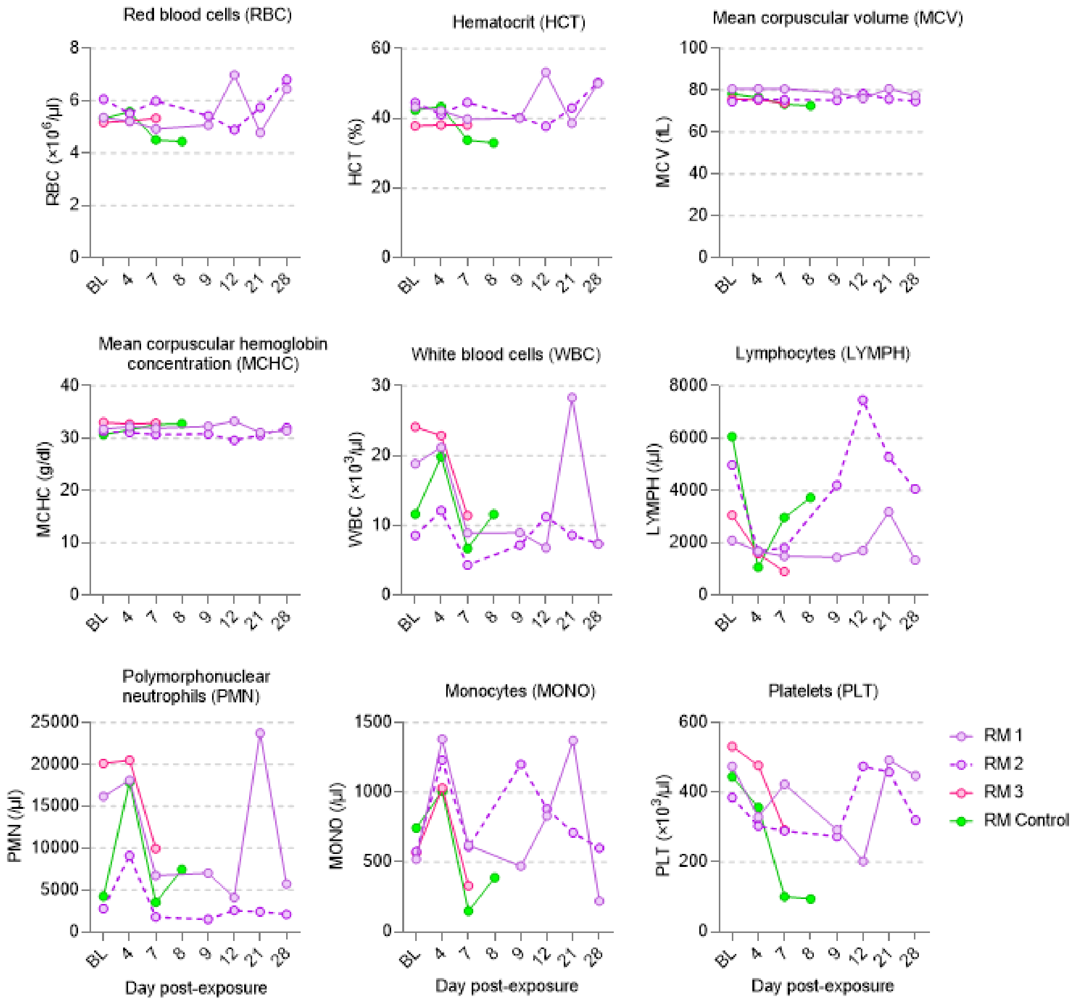

Supplementary Fig 1 Hematology analytes over time from anticoagulated whole blood of macaques exposed to Ebola virus (EBOV). Baseline (BL) values are averaged values, collected on Day -13 and Day -7 (prior to virus exposure on Day 0). All macaques that met euthanasia criteria or were found dead are indicated in magenta, survivors are indicated in purple, and controls are indicated in green.

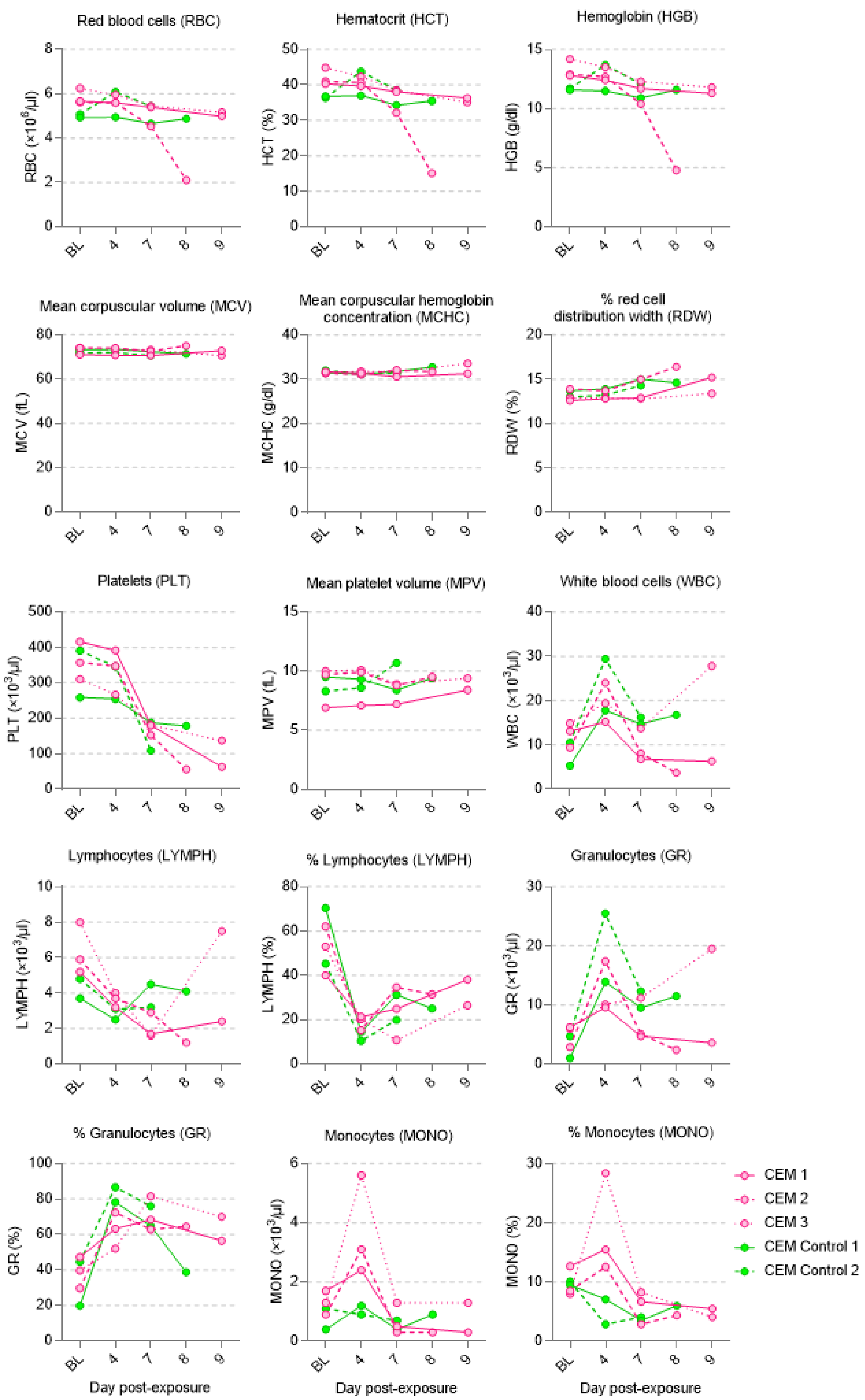

Supplementary Fig 2 Hematology analytes over time from anticoagulated whole blood of macaques exposed to Sudan virus (SUDV). Baseline (BL) values were collected on Day 0 prior

to virus exposure. All macaques that met euthanasia criteria or were found dead are shown in magenta, and controls are shown indicated in green.

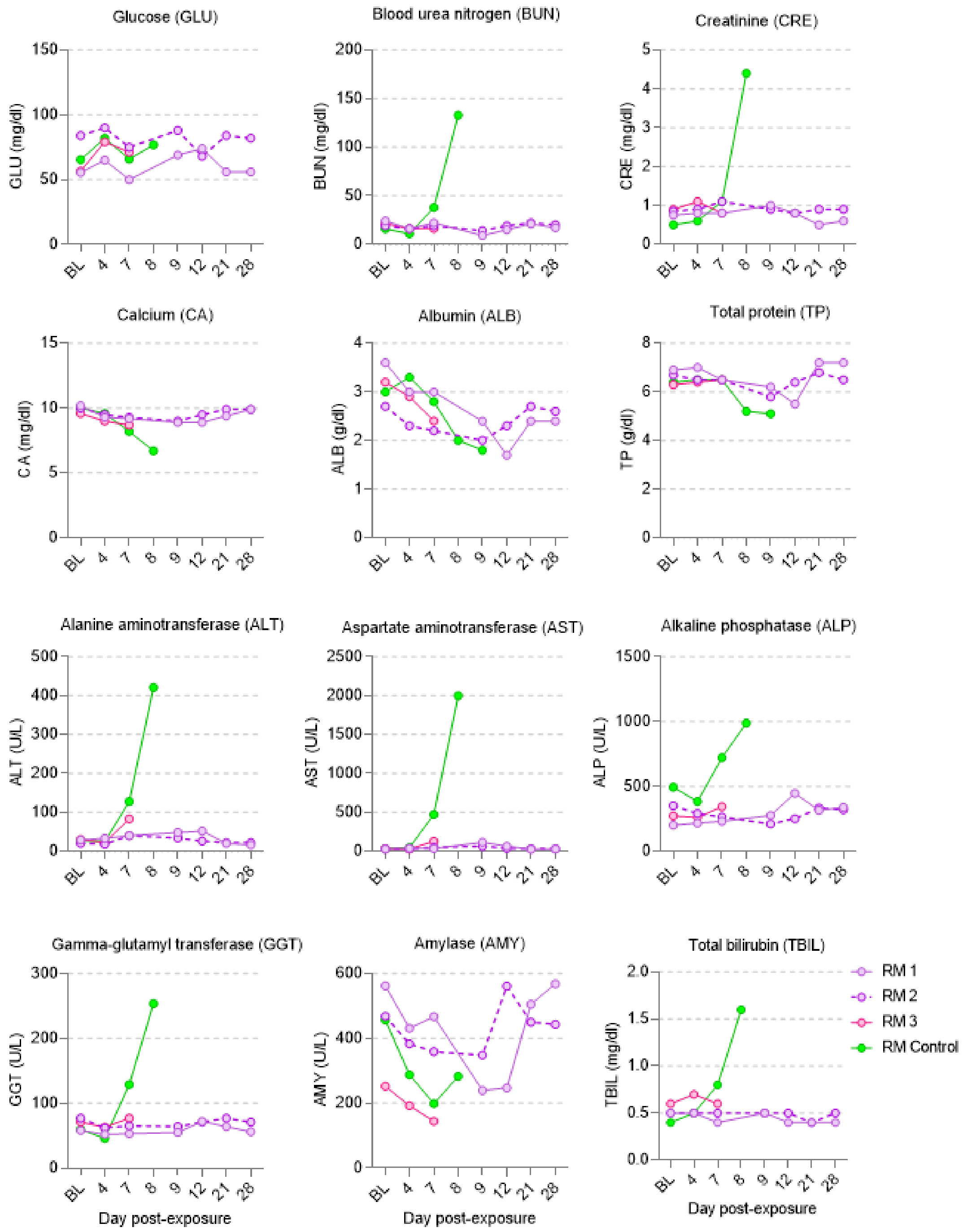

Supplementary Fig 3 Serum chemistry analytes over time of macaques exposed to Ebola virus (EBOV). Baseline (BL) values are averaged values, collected on Day -13 and Day -7 (prior to virus exposure on Day 0).

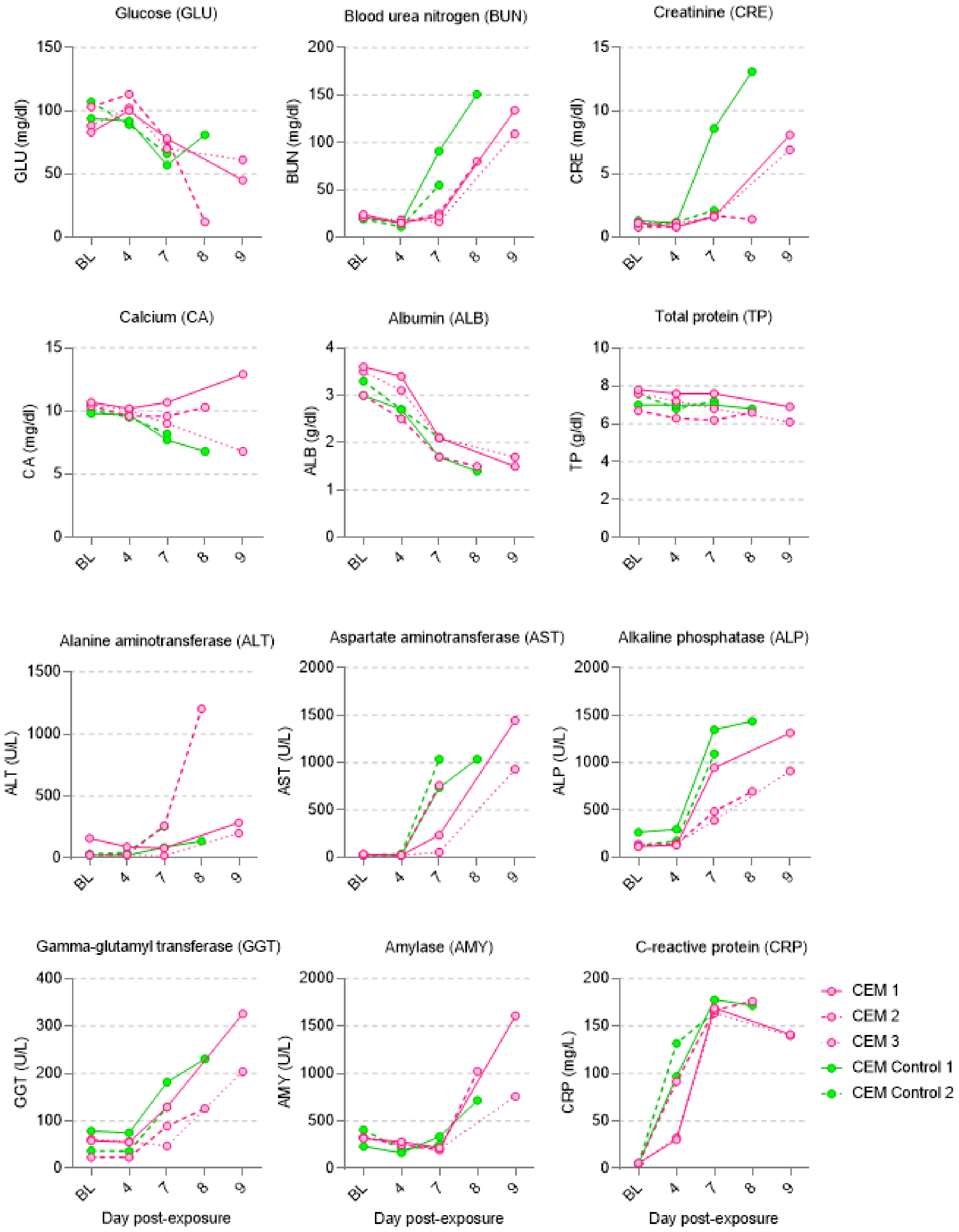

Supplementary Fig 4 Serum chemistry analytes over time of macaques exposed to Sudan virus (SUDV). Baseline (BL) values were collected on Day 0 prior to virus exposure.
